# The Role of Epigenetics in Autoimmune/Inflammatory Disease

**DOI:** 10.3389/fimmu.2019.01525

**Published:** 2019-07-04

**Authors:** Anna Elisa Andrea Surace, Christian M. Hedrich

**Affiliations:** ^1^Department of Women's and Children's Health, Institute of Translational Medicine, University of Liverpool, Liverpool, United Kingdom; ^2^Department of Paediatric Rheumatology, Alder Hey Children's NHS Foundation Trust Hospital, Liverpool, United Kingdom; ^3^Pädiatrische Rheumatologie, Klinik und Poliklinik für Kinder- und Jugendmedizin, Universitätsklinikum Carl Gustav Carus, TU Dresden, Dresden, Germany

**Keywords:** autoimmune, inflammation, epigenetic, chromatin, DNA methylation, inflammasome, psoriasis, lupus

## Abstract

Historically, systemic self-inflammatory conditions were classified as either autoinflammatory and caused by the innate immune system or autoimmune and driven by adaptive immune responses. However, it became clear that reality is much more complex and that autoimmune/inflammatory conditions range along an “inflammatory spectrum” with primarily autoinflammatory vs. autoimmune conditions resembling extremes at either end. Epigenetic modifications influence gene expression and alter cellular functions without modifying the genomic sequence. Methylation of CpG DNA dinucleotides and/or their hydroxymethylation, post-translational modifications to amino termini of histone proteins, and non-coding RNA expression are main epigenetic events. The pathophysiology of autoimmune/inflammatory diseases has been closely linked with disease causing gene mutations (rare) or a combination of genetic susceptibility and epigenetic modifications arising from exposure to the environment (more common). Over recent years, progress has been made in understanding molecular mechanisms involved in systemic inflammation and the contribution of innate and adaptive immune responses. Epigenetic events have been identified as (i) central pathophysiological factors in addition to genetic disease predisposition and (ii) as co-factors determining clinical pictures and outcomes in individuals with monogenic disease. Thus, a complete understanding of epigenetic contributors to autoimmune/inflammatory disease will result in approaches to predict individual disease outcomes and the introduction of effective, target-directed, and tolerable therapies. Here, we summarize recent findings that signify the importance of epigenetic modifications in autoimmune/inflammatory disorders along the inflammatory spectrum choosing three examples: the autoinflammatory bone condition chronic nonbacterial osteomyelitis (CNO), the “mixed pattern” disorder psoriasis, and the autoimmune disease systemic lupus erythematosus (SLE).

## Introduction

Autoimmune/inflammatory diseases are characterized by systemic or organ-specific inflammation resulting in tissue damage (1). Historically, autoimmune/inflammatory conditions were categorized into autoinflammatory vs. autoimmune disorders. Autoinflammatory disorders were defined by systemic or organ specific inflammation that occurs in the absence of high-titer autoantibodies and autoreactive lymphocytes. Autoimmune conditions, on the other hand, were classified as such disorders that are driven by the adaptive immune system and therefore characterized by the presence and pathophysiological involvement of autoantibodies and/or self-reactive lymphocyte populations (2). More recently, it became clear that the situation may be more complex and that only few monogenic disorders, such as e.g., cryopyrin-associated periodic syndromes (CAPS), primary type I interferonopathies, and other “monogenic” disorders can clearly be classified as primarily autoinflammatory in nature. This, however, can change over the course of disease. Tissue damage frequently results in cell death and subsequently the presentation of intracellular and nuclear components to adaptive immune cells, their activation and, as a consequence, in autoantibody production and/or self-directed lymphocyte responses (2). Based on this and the observation that some disorders initially show a mixed immunological pattern (e.g., psoriasis) that drive inflammation, the “inflammatory spectrum” has been proposed (3). Systemic autoimmune/inflammatory conditions can be stratified along the spectrum with monogenic autoinflammatory conditions at the one and “classical” autoimmune conditions at the other end of the spectrum. The paradigm of an inflammatory spectrum also allows to consider autoimmune/inflammatory conditions as dynamic processes in which molecular phenotypes can change and define variable clinical phenotypes, outcomes, and treatment responses (2–4).

Reflecting the constantly increasing number of known autoimmune/inflammatory conditions, aforementioned inter-individual variability in phenotypes, and outcomes (sometimes even between patients with the same diagnosis and/or between family members) (5), and the observation that initially distinct disorders may move along the inflammatory spectrum from e.g., a primarily autoinflammatory to an autoimmune phenotype [this can e.g., happen over time in systemic JIA and adult Still's disease (6, 7)], the molecular pathophysiology of autoimmune/inflammatory conditions is complex and only partially understood. To our current understanding, autoimmune/inflammatory diseases are based on monogenic disease causes (rare) or complex genetic predisposition (more common) that are subject to individual and environmental influences that define disease expression and/or individual phenotypes and outcomes (2, 4, 8–10). This becomes particularly clear considering “classical” autoimmune disorders in which genetically identical monozygotic twins can be discordant regarding the development of autoimmune/inflammatory conditions. Disease concordance rates between monozygotic twins range between 13% in multiple sclerosis (MS) (11), 20% in psoriasis (12), 24% in type 1 diabetes (T1D) (13), and 14.3–40% in systemic lupus erythematosus (SLE) (14–16). These observations resulted in the hypotheses that (i) disease-causing single gene mutations in rare autoinflammatory disorders cause disease-onset, but do not necessarily define individual outcomes, and that (ii) genomic variants define disease susceptibility in genetically complex disorders, but disease expression, individual phenotypes, and outcomes are instigated and defined by additional factors, including epigenetics (17).

Epigenetic events are gene regulatory mechanisms that control the accessibility of chromatin to transcriptional regulatory factors, thereby tuning gene expression without changing the underlying DNA sequence (18). Epigenetic events can be influenced by the environment, are dynamic but also heritable and are in the end responsible for significant variation between cells and tissues in one organism, independent of the identical genotype between all diploid cells (19). Epigenetic mechanisms include DNA methylation, post-translational modifications to histone proteins, and non-coding RNA expression.

### DNA Methylation and DNA Hydroxymethylation

**Figure 1:**
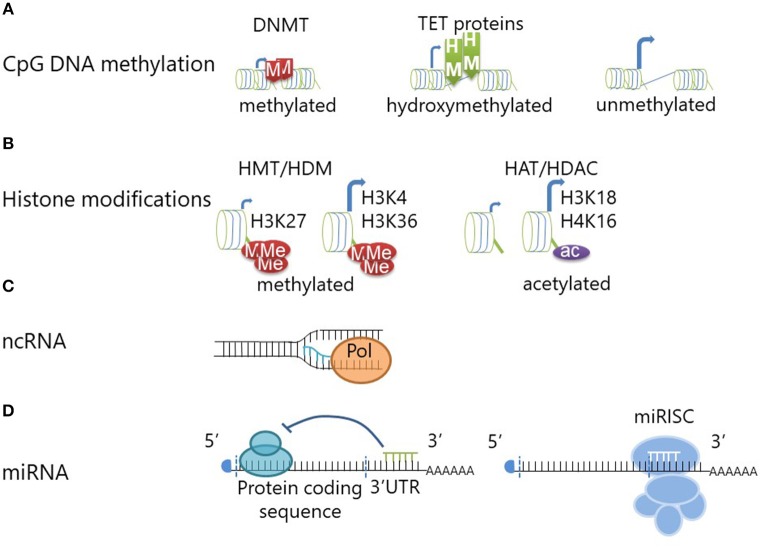
Epigenetic modifications regulate gene transcription and translation. **(A)** DNA methyltransferase (DNMT) enzymes maintain or generate (*de novo*) DNA methylation at CpG dinucleotides. DNA methylation confers repression of gene expression through reduced transcription factor accessibility. DNA Hydroxymethylation is achieved through oxidation of methylated CpG DNA and mediated by Ten-eleven translocation methylcytosine dioxygenase (TET) proteins. DNA hydroxymethylation defines an “open” chromatin structure which allows for gene transcription (similar to unmethylated CpG DNA). **(B)** Histone methyltransferases (HMT) can add (one to three) methyl groups to histone amino termini. Depending on the exact molecular location and the degree of histone methylation, this can lead to chromatin compaction or decompaction. Methylation of histone H3 at lysine 27 (H3K27) will lead to chromatin compaction and transcriptional repression, while methylation at H3 lysine 4 (H3K4) and H3K36 mediates “opening” and increases transcription. Histone demethylases (HDM) can counteract this by removing the methyl groups. Histone acetylation is mediated by histone acetyltransferases (HAT) and can be reversed by histone deacetylases (HDAC). Histone acetylation is associated with chromatin decompaction and transcription of genes. **(C)** The transcription of non-coding RNA from intergenic or intronic regions can promote coding mRNA transcription by providing an open chromatin formation. **(D)** Short micro-RNAs (miRNA) can mediate transcriptional repression through inhibition of the ribosome when binding to the 3'UTR region of mRNAs. Furthermore, miRNAs can induce degradation of the mRNA through initiation of the miRISC complex.

The addition of a methyl group onto the 5′carbon position of cytosine in cytosine-phosphate-guanosine (CpG) dinucleotides can potently reduce accessibility to DNA for transcription factors and RNA polymerases, and thereby repress transcription (see Figure 1A). DNA methyltransferase (DNMT) enzymes are responsible for maintaining methylation. DNA re-methylation is essential during cell division to copy the epigenetic code to the daughter cell generation, but also *de novo* methylation to silence previously active genes plays a role in gene regulation. DNMT1 and DNMT2 are responsible for re-methylation of DNA during cell division (20, 21), while DNMT3a and 3b introduce new methyl groups to previously unmethylated DNA (22). However, the situation may be more complex and discrimination between maintenance and *de novo* DNMTs may be an unjustified oversimplification; e.g., DNMT1 is indeed involved in both methylation of daughter strands during cell division and *de novo* methylation of regulatory regions (10).

DNA methylation can be changed by Ten-eleven translocation methylcytosine dioxygenase (TET) proteins which convert methyl- to hydroxymethylcytosines (23). Thus, DNA hydroxymethylation is considered an intermediate on the way from heavy DNA methylation of transcriptionally repressed genes to a demethylated state at open and transcriptionally active chromatin (24). Hydroxymethylated CpG sites are protected from DNMTs and therefore from DNA methylation (25). The absence of TET proteins and therefore inhibition of hydroxyl-conversion, does, however, not necessarily result in higher DNA methylation levels. Thus, hydroxymethylation is considered an independent and also stable epigenetic event (23). Indeed, DNA hydroxymethylation is considered an activating epigenetic mark and coincides with increased gene expression when compared to methylated DNA (24) (see Figure 1A).

### Histone Modifications

To three-dimensionally organize its composition and accessibility to the transcriptional complex, DNA is wrapped around histone protein complexes, octamers consisting of two copies of each histones H2A, H2B, H3, and H4. Histone proteins can be modified at their N-terminal amino residues, which mediates changes in their electrical charge and thereby defines accessibility of chromatin to the transcriptional complex (8). A number of histone modifications has been reported, including methylation, citrullination, or acetylation (e.g. see Figure 1B). Examples for “silencing” histone modifications include histone 3 lysine 9 di-methylation (H3K9me2), H3K9 tri-methylation (H3K9me3), or H3K27me3, whereas histone 4 lysine 16 acetylation (H4K16ac), H3K4me3, H3K18ac, H3K27ac, or H3K56ac are markers of active transcription (10, 26–28).

Of note, histone modifications, and CpG methylation are connected through methyl-CpG-binding proteins (MBD) (29). These proteins recruit both histone deacetylases and methyltransferases which cause silencing through histone modification (29, 30). Moreover, histone tail modifications can allow or prohibit binding of DNMT3 (31).

### Non-coding RNAs

Non-coding RNAs can be derived from both introns and intergenic regions. They include microRNA, short interfering RNA, and long non-coding RNA among others. Non-coding transcripts in intergenic regions regulate the chromatin accessibility during transcription by maintaining an open chromatin structure (32) (see Figure 1C). Furthermore, intergenic transcription may mediate interactions between promoter regions and distal enhancers, sometimes between chromosomes (33). While these concepts are promising and of potentially central interest in the context of dysregulated inflammatory responses and autoimmune disease, data on the involvement of long non-coding RNA expression in immune homeostasis is incompletely understood and remains subject to speculation.

More scientific focus has been put on deciphering the effects of microRNAs (miRNA), which contribute heavily to fine regulation of gene expression. While discussed controversially by some authors, miRNAs fulfill the definition of epigenetic events (define cellular phenotypes through the regulation of gene expression while not affecting the underlying DNA sequence, they are heritable but also inducible and reversible) (9). Micro-RNAs are 21–23 bases long and target mRNAs mainly at the 3′ untranslated region (3′UTR). Interaction with mRNA leads to cleavage and degradation, thereby preventing translation (see Figure 1D) and inducing translational arrest (34). On top of being considered an epigenetic event, miRNAs regulate other epigenetic events, e.g., through targeting DNMTs and thereby mediating DNA hypomethylation. Micro RNAs-29,−29b, and−143 interfere with the expression of DNMT3a and−3b and indirectly also DNMT1 (35–37). Currently, ~2,000 human miRNAs have been described (38). The involvement of miRNAs in human disease has been established, including inflammatory disease, and cancer. Some miRNAs have multiple targets and functions and can therefore be seen as “multivalent” agents that regulate the expression of several proteins and multiple miRNAs can interfere with the expression of single genes (38). Variations of miRNA expression between ethnic populations (which also exhibit variable risk, phenotypes, and outcomes in autoimmune/inflammatory disease) have made them interesting candidates in the search for molecular pathomechanisms in inflammatory conditions. Potential importance in the pathophysiology of autoimmune disease is suggested by their involvement in the regulation of up to 80% of human genes (39).

In the following, we will discuss the contribution of epigenetic alterations to systemic autoimmune/inflammatory disorders along the inflammatory spectrum choosing three examples: (i) inflammasome-associated autoinflammatory diseases (namely cryopyrin-associated periodic syndromes; CAPS and chronic nonbacterial osteomyelitis; CNO), (ii) the mixed-pattern disease psoriasis, and (iii) the “prototypical” autoimmune disorder Systemic Lupus Erythematosus (SLE). This work is not an all-inclusive review of the literature available, but rather aims at introducing concepts, and mechanisms behind epigenetic events and their contribution to autoimmune/inflammatory conditions along the inflammatory spectrum.

## Epigenetic Events in Inflammasome-Associated Autoinflammatory Disease

Autoinflammatory disorders are characterized by systemic or organ specific inflammation in the absence of autoreactive lymphocytes and high-titer autoantibodies. Among the first autoinflammatory conditions genetically identified were inflammasome-associated disorders that are characterized by increased activation of the NLRP3 inflammasome, a cytoplasmic multi-protein complex that assembles in response to contact with “danger signals” and mediates the activation of caspase-1 (40). Caspase-1 cleaves inactive pre-IL-1β that is stored in the cytoplasm into its active form IL-1β, which is then released from the cell and induces strong down-stream pro-inflammatory responses (41, 42). Furthermore, caspase-1 mediated cleavage of gasdermin D (a pore forming protein) leads to inflammatory cell death which is referred to as pyroptosis (43). Prototypical representatives of this group are rare monogenic conditions following “Mendelian” traits of inheritance and include (but are not limited to) the TNF receptor-associated periodic syndrome (TRAPS), familial Mediterranean fever (FMF), and CAPS (44). These conditions (at least at disease-onset) lack classical autoimmune features (autoantibodies, autoreactive B and T cells) that are observed in autoimmune diseases, such as SLE.

Mutations in the cytoplasmic danger sensor NLRP3 cause CAPS. Cryopyrin-associated periodic syndromes cover a spectrum of disorders with different severity, including the least severe form familial cold autoinflammatory syndrome (FCAS), Muckle-Wells syndrome, and the most severe Neonatal-onset Multisystem Inflammatory Disease (NOMID). All of these can be caused by identical mutations in the NLRP3 gene and it remains currently unknown why some patients (even within one family) develop FCAS while others experience NOMID. CAPS patients display spontaneous activation of the NLRP3 inflammasome and increased IL-1β release which clinically manifests with skin, joint, and central nervous system inflammatory disease (45). Of note, only about 50–60% of patients with NOMID exhibit mutations in the NLRP3 gene leaving the rest currently unexplained. Low-level mosaicism in “mutation-negative” CAPS patients can be detected using new generation deep sequencing techniques and explain some additional cases. However, unexplained cases with unknown molecular pathophysiology remain (46).

Epigenetic mechanisms are involved in the pathophysiology of CAPS. Lesional and non-lesional skin biopsies from CAPS patients unveiled down-regulation of 813 genes which included histone proteins e.g., *HIST2H2AC*, histone modifying genes e.g., *HDAC1, HDAC2, SUMO1*, and genes connected to methylated DNA e.g., *MBD2* (47). Histone deacetylase enzymes (HDACs) remove acetyl groups from amino-termini of histone proteins and therefore affect chromatin arrangement (48). The suggestion that CpG DNA methylation may be altered in CAPS coincides with changes in DNA methylation observed in monocytes from CAPS patients as compared to healthy controls. While the methylation status of *IL1B, NLRC5, PYCARD, AIM2*, and *CASP1* remained the same in healthy controls after stimulation, CAPS patients show strong demethylation of those genes in monocytes and monocyte-derived macrophages in response to IL-1β (49). It is worth mentioning that IL-1 blocking treatment reversed these effects, indicating that increased IL-1β activation, and release may prime DNA demethylation which could maintain and/or amplify inflammation.

However, data on the involvement of epigenetic events in the pathophysiology of CAPS remain somewhat controversial. While Aubert et al. described downregulation of transcripts, Vento-Tormo et al. (49) described DNA demethylation at inflammasome-associated genes, which suggests increased gene expression. However, the latter group has investigated only a small group of genes which were not included in the study of Aubert et al. This indeed suggests that DNA methylation (and other epigenetic events) are complex, tissue-, gene-, and element-specific and may vary significantly between disorders and individuals. Furthermore, it is important to note that, while a number of genes are down-regulated in CAPS, inflammasome-related genes [which exhibit reduced DNA methylation in the study by Vento-Tormo et al. (49)] are upregulated, underscoring their role as main drivers in the disease. Additionally, transcriptomics data from Aubert et al. showed that miRNAs are generally upregulated in lesions of NOMID patients compared to non-lesional skin or healthy controls, which may (at least) partially be responsible for decreased mRNA detection in the same study (47).

Several miRNAs upregulated in CAPS target NLRP3 and associated molecules. The NLRP3 sensor component of the inflammasome can be regulated by miRNAs, including miR-7, miR-20, miR-133b, or miR-223 (50). Binding of these miRNAs to 3'UTR regions leads to reduced NLRP3 expression and IL-1β release (51). Both miR-7 and miR-133b are increased in non-lesional skin biopsies from NOMID patients as compared to healthy controls. Since this was even more pronounced in lesional skin biopsies, authors argue that miRNA expression may represent a regulatory mechanism counteracting inflammasome activation, which, however, may not be sufficient in pathologically over-active situations, such as CAPS (47). miR-203 is another miRNA upregulated in skin lesions of CAPS patients which exerts pro-inflammatory effects in the skin. It downregulates the suppressor of cytokine signaling 3 (SOCS-3) by binding to the 3'UTR region of its mRNA (52). SOCS-3 protein is important to attenuate IL-6 mediated activation of the transcription factors signal transducer and activator of transcription (STAT)3 and STAT1 and inhibition of this negative feedback loop may allow for prolonged inflammatory activity (53). This is in contrast to findings reported by Aubert et al. who determined (not statically significant) increased SOCS-3 mRNA expression in lesional skin biopsies from CAPS patients. On the other hand, lower levels of SOCS-3 mediated through increased levels of miR-203 may allow for increased STAT3 activation and subsequent IL-6 signaling, which is a hallmark of CAPS/NOMID (47).

Taken together, though incompletely understood, epigenetic mechanisms may play an essential role during disease progression in CAPS by increasing the production of inflammasome components. Epigenetic events may, at least partially, explain inter-individual differences in disease severity within families and promise potential as future disease biomarkers and/or targets for individualized therapeutic interventions.

Another inflammasome-associated autoinflammatory disease is CNO. It mostly affects children and adolescents and is characterized by spontaneous bone inflammation that can result in pain, bone deformity and even fractures. While some patients exhibit monofocal and sometimes timely limited and monophasic disease, others develop chronically active or recurrent bone inflammation at multiple sites. In such cases, the term chronic recurrent multifocal osteomyelitis (CRMO) is used (54). A subset of patients exhibits additional inflammatory symptoms, including psoriasis, and palmoplantar pustulosis, severe acne and/or inflammatory bowel disease. While the exact molecular pathophysiology of CNO remains unclear, it became apparent that reduced expression of immune-regulatory cytokines IL-10 and IL-19 as well as increased NRLP3 inflammasome assembly are centrally involved (55, 56). Despite *IL10* promoter polymorphisms that are meant to result in increased IL-10 expression, patients with CNO exhibit reduced IL-10 expression in monocytes in response to Toll-like receptor (TLR-)4 activation with lipopolysaccharide (LPS). This has been linked with failure to activate mitogen activated protein kinases (MAPK) extracellular signal regulated kinase (ERK)1 and 2 in monocytes. This in turn results in reduced phosphorylation of histone 3 serine 10 (H3S10P) at the *IL10* and *IL19* promoter regions (57). IL-10 and its homolog IL-19 are immune regulatory cytokines that undergo regulation through epigenetic events. For example, it has been described, that phosphorylation of histone H3 (mouse derived macrophages) allows for increased expression of IL-10 (58). Furthermore, strongly correlated tissue/cell specific co-regulation has been described for *IL10* and *IL19*. Dependent on the cell type, variable CpG sites are methylated contributing to differential expression of cytokines (59–61). In addition to effects on epigenetic marks in monocytes from CNO patients, reduced ERK1/2 activation prohibits activation of the transcriptional regulatory factor signaling protein (Sp-)1 which results in reduced nuclear translocation and recruitment to *IL10* and *IL19* (56). Together, reduced H3S10P (an activating epigenetic mark) and impaired Sp-1 phosphorylation result in altered IL-10 and IL-19 expression in monocytes from CNO patients (57).

*IL10, IL19*, and the pro-inflammatory IL-10 family cytokine *IL20* are located in the *IL10* cluster on chromosome 1 (33). In contrast to IL-10 and IL-19, the pro-inflammatory cytokine IL-20 is expressed at increased levels in monocytes from CNO patients in response to TLR-4 stimulation with LPS. While IL-20 expression is not dependent on Sp-1, reduced DNA methylation at the *IL20* promoter may favor gene expression. Of note, both the *IL10* and *IL19* promoters show no difference in CpG DNA methylation between CNO patients and healthy controls (55). This underscores the complex interplay between epigenetic modifications and effects of alterations to their composition.

Reduced expression of IL-10 and IL-19 contribute to inflammation by allowing increased expression of inflammasome components and inflammasome assembly resulting in IL-1β secretion (55, 62). As also suggested for CAPS by Vento-Tormo et al. (49), increased expression of inflammasome components can similarly be linked to reduced DNA methylation at genes encoding for inflammasome components (*NLRP3* and *PYCARD*) in CNO monocytes (62). Whether DNA demethylation is a result of reduced expression of immune-regulatory IL-10 and IL-19 remains elusive. Another possible contributor to DNA demethylation may be the aforementioned reduced activation of ERK1/2 in monocytes from CNO patients (57). Indeed, DNA demethylation of T cells from SLE patients is a result of reduced MAPK activation, which centrally contributes to the pro-inflammatory effector phenotype of these cells (49, 50).

**Figure 2:**
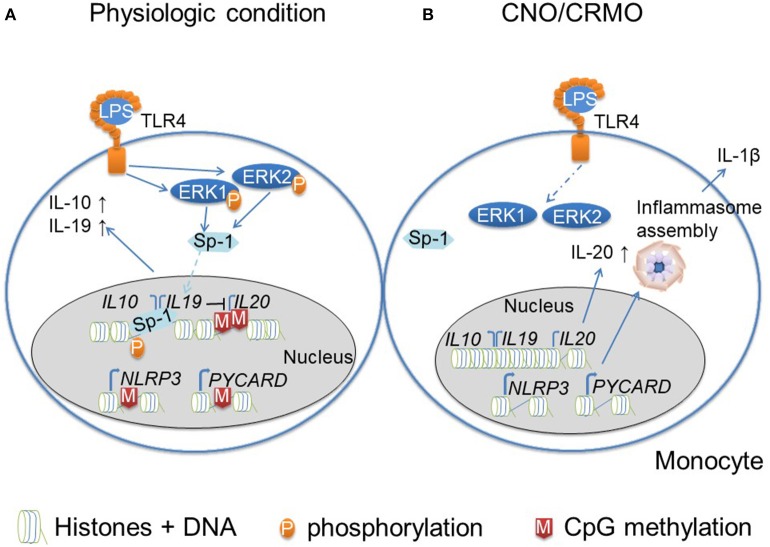
Monocytes from CNO/CRMO patients are epigenetically primed for inflammation. **(A)** In response to TLR4 activation (with lipopolysaccharide; LPS) monocytes from healthy individuals phosphorylate mitogen activated protein kinases (MAPK) extracellular signal reactive kinases (ERK)1 and 2. Kinases activate the transcriptional regulatory factor signaling protein Sp-1, which results in its translocation into the nucleus. Furthermore, ERK1/2 contribute to histone H3 phosphorylation at serine residue 10 (H3S10) resulting in an open chromatin structure at *IL10* and *IL19*. These events together result in trans-activation of IL10 and IL19. Immune regulatory cytokine expression (IL-10 and IL-19) negatively affect the expression of the pro-inflammatory *IL10* cytokine family member IL-20. Furthermore, the *IL20* promoter is controlled by CpG DNA methylation. Inflammasomes are multi-protein complexes that become activated in response to “danger signals.” Furthermore, the expression of inflammasome components (NLRP3 and ASC/PYCARD) is regulated by epigenetic events. Promotors of *NLRP3* and *PYCARD* are controlled by CpG DNA methylation. **(B)** In monocytes from CNO/CRMO patients, ERK1/2 activation in response to LPS stimulation is impaired which results in reduced Sp-1 activation and nuclear shuttling, and decreased H3S10 phosphorylation at *IL10* and *IL19* resulting in impaired gene expression. Reduced levels of immune regulatory cytokine expression allows for higher expression of the pro-inflammatory cytokine IL-20. Furthermore, impaired IL-10 and IL-19 expression promoted expression of inflammasome components, and reduced CpG DNA methylation of the *PYCARD* and NLRP3 genes further increase pro-inflammatory molecule expression. Thus, epigenetic events are involved in the molecular pathophysiology of CNO/CRMO.

Taken together, these observations strongly suggest a pathophysiological (CNO, CAPS) or disease-amplifying or modifying (CAPS) role for epigenetic events in inflammasome-associated autoinflammatory conditions (summarized in Figure 2). This makes epigenetic alterations promising candidates in the search for biomarkers for individual outcomes, and/or targets for disease-modifying interventions.

## Epigenetic Events in the Mixed Pattern Disease Psoriasis

Psoriasis is a systemic autoimmune/inflammatory condition that manifests with skin involvement. A subset of patients develops additional symptoms and organ involvement, such as arthritis. Psoriatic arthritis (PsA) is characterized by sometimes treatment resistant, chronic progressive and destructive arthritis. Approximately 0.27% of patients with adult-onset psoriasis will develop PsA per year (63). Considering the inflammatory spectrum, psoriasis exhibits features of a mixed-pattern disease based on stage and phenotype specific involvement of mediators of innate and/or adaptive immune effectors. Especially in early stages of the disease, but also during flares, the innate immune system (neutrophils, monocytes/macrophages, mast cells, and dendritic cells) is heavily involved in skin infiltrates. At this stage, pro-inflammatory molecules including IL-1β, TNF-α, and Interferon (IFN)-γ are key drivers of inflammation (64). The innate molecular caspase recruitment domain family member (CARD)14 resembles a key element linking innate and adaptive immune mechanisms. Variants in *CARD14/PSORS2* are associated with familial (mutations) and multifactorial (nucleotide polymorphisms) psoriasis (65, 66). Genetic variants contribute to the expression of pro-inflammatory cytokines including TNF-α, IL-6, and IL-8, colony stimulating factor (CSF)2 and matrix metallopeptidase (MMP)9 among others. This is an effect of increased NFκB signaling (66), subsequently increased pro-inflammatory molecule expression and the attraction of immune cells e.g., neutrophils through IL-8 (67). Furthermore, the mixed pattern character of psoriasis involves the presence of autoimmune features and the presence and activation of the adaptive immune cells. Effector Th17 cells are implicated in psoriasis and therapeutic targeting of IL-17 can alleviate symptoms (68). In later stages of psoriasis, effector Th1 lymphocytes play an increasing role in the disease (64). Th1 cells produce pro-inflammatory cytokines including IL-2 and IFN-γ. Effector T cell populations are believed to be a product of type I interferon expression (IFN-α, IFN-β) derived from plasmocytoid dendritic cells (69). Indeed, IFN-γ is increased in psoriatic plaques and treatment of other diseases with IFN-γ can trigger psoriasis and psoriatic arthritis or induce flares (69, 70).

### Epigenetic Changes in Immune Cells of Psoriasis Patients

DNA methylation plays a role in promoting the pro-inflammatory immune cell phenotype of psoriasis. Peripheral blood mononuclear cells (PBMC) from patients with chronic plaque psoriasis show significantly reduced levels of DNA methylation across the genome (71). In line with these observations, CD4^+^ T cells from psoriasis patient's exhibit reduced DNA methylation at promoter regions of genes on all chromosomes (72). Recently, Brandt et al. reported reduced DNA methylation at a distal enhancer element of the *IFNG* gene (encoding for interferon-γ) in effector CD4^−^CD8^−^CD3^+^TCR^+^ (“double negative”; DN) T cells (73). Furthermore, authors demonstrated that DN effector T cell infiltrate inflamed tissues and may therefore contribute to inflammation and damage. Indeed, increased IFN-γ expression in DN T cells may be of pathophysiological relevance, since plaques of psoriasis patients exhibit strongly Th1 driven skin inflammation (74). In contrast to these observations in PBMCs and total CD4^+^ T cells, naïve CD4^+^ T cells from psoriasis patients exhibit methylation profiles that are mostly comparable to healthy controls (75).

Currently, studies investigating histone modification in immune cells from psoriasis patients are sparse. One study, investigating histone marks in PBMCs from psoriasis patients delivered reduced levels of histone H3 and H4 acetylation and increased H3K4 methylation (76). Histone methylation and acetylation were not measured at specific genes, but as a global marker within all PBMCs and did not decipher between specific methylation or acetylation patterns (e.g., mono- or trimethylated) or between specific loci on chromosomes. Global histone acetylation and methylation patterns were similar in PBMCs from psoriasis and PsA patients. Of note, while H3K27 methylation did not differ between healthy controls and untreated psoriatic disease patients, there was a significant increase in this histone marker after treatment, only for patients who responded to biologics. In contrast, non-responders had no change in H3K27 methylation after treatment compared to before treatment. In psoriasis patients, excluding those with arthritis, reduction in H3K4 methylation after 3 months of treatment associated with treatment response (76). Of note, H3K27 demethylation in CD4^+^ T cells is involved in effector T cell generation in autoimmune disease (77) and drives Th17 differentiation (78). Thus, increased H3K27 methylation may contribute to immune modulation and disease control in treatment responders. This suggests that H3 and H4 acetylation as well as H3K4 methylation may be utilized as future biomarkers for psoriasis, and that H3K27me3 may have potential in the search for biomarkers of treatment response.

Indeed, Th17 phenotypes appear to be central to skin inflammation and tissue damage in psoriasis. An imbalance between CD4^+^ Tregs and Th17 cells has been described in psoriasis (79). However, IL-17A production is not limited to CD4^+^ T helper subsets. Also DN T cells produce this inflammatory cytokine and (as mentioned above) infiltrate the skin of patients with plaque psoriasis (80). In psoriasis, DN T cells are characterized by increased surface expression of the programmed cell death 1 (PD1) co-receptor, a regulatory “immune checkpoint” that is involved in the termination of inflammatory responses and immune homeostasis (73, 81). Furthermore, DN T cells are incapable of proliferation, which may be a regulatory response to their self-reactive nature or a reflection of their over-activated and/or terminal differentiation state. While, no data on IL-17 expression from DN T cells in psoriasis has been published yet, aforementioned reduced CpG DNA methylation of a distal enhancer element of *IFNG* underscores their effector character and potential involvement in psoriasis (73).

In addition to DNA methylation and histone modifications, non-coding RNAs are involved in the imbalance between regulatory and effector T cells in psoriasis. The expression of miR-210 is increased in CD4^+^ T cells from psoriasis patients and negatively affects FOXP3 expression, thereby inhibiting immune regulatory functions (82).

Though associated with poor outcomes, the molecular pathophysiology of PsA is largely unstudied and few data exist on the involvement of epigenetics. One study investigated miRNA profiles of PBMCs from PsA patients with inactive or active disease (83). While 22 miRNAs were differentially expressed in PBMCs from active patients, ten were specific to inactive, and 12 were specific to PsA independent of disease status. Investigation of the role of differentially expressed miRNAs may contribute to a better understanding of the molecular pathophysiology of PsA. Interestingly, downregulated miRNAs in PBMCs from patients with active disease for example would target SPP1 and TNF (83). SPP1 encodes for osteopontin, a protein involved in bone remodeling, a neutrophil chemoattractant and a facilitator of T cell activation (84). TNF-α is a potent pro-inflammatory cytokine usually associated with innate immune responses. This underscores the activation of innate and adaptive immune responses in psoriasis and psoriatic arthritis, and suggests that non-coding RNAs are centrally involved in the pathophysiology and accrual of damage in PsA (83).

Though research strongly focuses on the involvement of adaptive immune responses in the pathophysiology of psoriasis, the importance of the innate immune system, especially in early stages should not be neglected [as described above (85)]. The pathophysiological significance of these is underscored by the associations of genetic variants in innate immune response related genes with psoriasis. Polymorphisms in inflammasome and NFκB pathway associated genes, such as *CARD14, NLRP1*, and *NLRP3*, increase the individual's risk to develop psoriasis (65, 85–87). Importance of the inflammasome and the innate immune system shown in genetic studies is in contrast to the current lack of epigenetic research about them.

### Epigenetic Changes in Skin Cells From Psoriasis Patients

Psoriasis is characterized by variable clinical pictures and disease outcomes. Thus, it is not surprising that more recently preliminary data became available indicating a role of non-immune cells orchestrating and directing self-reactive immune responses in psoriasis (88). Indeed, the interplay between immune and stroma cells may contribute to a better understanding of why certain organ system but not others are affected in individual patients.

Affected skin collected from psoriasis patients exhibits globally reduced levels of DNA methylation (71). Another study demonstrated that skin biopsies from psoriasis patients differ in DNA methylation of over 1000 CpG sites when compared to skin from healthy individuals, while <30 CpGs differ regarding their methylation status between affected and unaffected skin from patients (89). Comparing unaffected skin from patients to skin obtained from healthy individuals, only 15 CpG sites exhibited differential DNA methylation. While a large number of regions did not show significant differences, their methylation levels ranged between the levels detected in healthy and affected psoriatic skin. Genes with reduced DNA methylation include *GALR1, GPR26, ZNF454, ZNF540, NEF3, RGS7, MLF1*, and *NRIP2*, none of which, however, are known to functionally relate to immune activation or autoimmune/inflammatory disease (89). Only the hypomethylated MLF1 gene had previously been reported to be expressed at higher levels in psoriasis. However, immunological functions have not been suggested (90). Another gene that is differentially methylated in psoriasis patients could be of significant interest. *PDCD1LG2*, hypermethylated in non-lesional skin of psoriasis patients when compared to healthy controls (89). It encodes for PD1 Ligand 2, a ligand for the aforementioned PD1 surface co-receptor which is elevated on effector DN T cells (73). While the aforementioned alterations to DNA methylation in skin cells are intriguing, no data on mechanisms involved are currently available. Thus, it is at least possible that disease-associated changes in DNA methylation may be due to the infiltration of immune cells and chronic inflammation rather than primarily responsible for skin involvement and damage (71).

Data on altered histone modifications of skin cells in psoriasis and their involvement in the pathophysiology are even sparser. Lesional skin biopsies from psoriasis patients show increased expression of HDAC1 when compared to controls (91). Besides the findings of Tovar-Castillo et al. (91), hypomethylation of the HDAC1 gene has also been described in affected psoriatic skin compared to healthy controls (89). This suggests reduced histone acetylation, which, however, was not investigated in this study. Inhibition of HDAC proteins results in reduced IL-17 production and the promotion of regulatory T cell phenotypes (79). Increased expression of HDAC1 and IL-17 in psoriasis suggests that HDAC1 may be a suitable target of future therapeutic interventions. However, currently, “epigenetic treatments” with HDAC inhibition remain a controversial topic (92), since (i) the primary involvement of HDACs in psoriasis is not sufficiently proven, and (ii) currently available agents globally inhibit HDAC activity and may cause significant and severe treatment-associated side effects.

Expression profiles of non-coding RNAs show similarities between inflammatory skin conditions. Psoriasis and atopic eczema share altered expression of ten miRNAs that are not expressed in the skin of healthy controls, five of which are increased (miR-20a,-146a,-17.5p,-21, and-106) and five of which are decreased (miR-122a,-133a,-133b,-326, and-215) (52). Exact targets and downstream effects are not fully understood for many of those miRNAs, at least for miR-21 it has been suggested that it contributes to the increased presence of T cells in psoriatic skin lesions through the suppression of apoptosis. Mechanisms behind this, however, remain to be investigated (93). Increased expression of miR-203 in keratinocytes from psoriasis patients inhibits SOCS3 (as described above for CAPS patients) (52). As a consequence, reduced SOCS3 expression leads to increased activation of STAT3, which favors pro-inflammatory cytokine expression (including IL-17A) (94).

In addition to the adaptive immune system and processes involved in keratinocyte regulation, also innate immune responses are dysregulated in psoriasis. They contribute to the pathophysiology of “innate forms” or psoriasis and/or disease flares in plaque psoriasis. miR-31, is overexpressed in keratinocytes from skin lesions of psoriasis patients. It contributes to the activation of NFκB, thereby inducing the production of early mediators of inflammation in psoriasis, namely IL-1β, CXCL1,-5, and-8. These cytokines are produced in human keratinocytes in the presence of TNF-α and inhibition of miR-31 reduces mRNA and protein expression of CXCL1,-5, and-8 (IL-1β data only available for the mRNA level). The direct target of miR-31 is serine/threonine kinase 40 which is responsible for dampening NFκB signaling, thereby reducing inflammatory cytokines such as CXCL1,-5,-8, and IL-1β (95). These effector molecules contribute to the chemotaxis of immune cells, and local as well as systemic inflammation (2, 67). Thus, miRNA-31 links innate and adaptive mechanisms in the mixed pattern disease psoriasis. Another example for increased innate immune activation in psoriatic skin is the increased expression of caspase 1 and IL-1β mRNA, and AIM2 inflammasome activation (96, 97).

Additional evidence for the involvement of miRNAs in pathological activation of innate immune responses in psoriasis comes from animal models. The imiquimod model of psoriasis in mice is characterized by increased protein expression of NLRP3 that positively correlated with miR-155 expression in skin lesions when comparted to unaffected skin. Overexpression of miR-155 in HaCaT keratinocytes results in increased NLRP3 and caspase-1 expression in response to priming with lipopolysaccharide. Authors concluded that this indicates positive effects of miRNA-155 on inflammasome activity and that this mechanism may centrally be involved in innate immune activation in psoriatic skin (98).

The involvement of epigenetic changes in keratinocytes are not limited to immune dysregulation, but also involved in the induction of keratinocyte proliferation. Reduced miR-125b expression in epidermal layers of psoriasis patients correlates with increased proliferation. This may be pathophysiologically meaningful, since miR-125b can suppress fibroblast growth factor receptor 2 (FGFR2), a protein that stimulates keratinocyte proliferation. Thus, reduced miR-125b can no longer sufficiently control proliferation of keratinocytes contributing to psoriasis typical hyperkeratosis (99).

**Figure 3:**
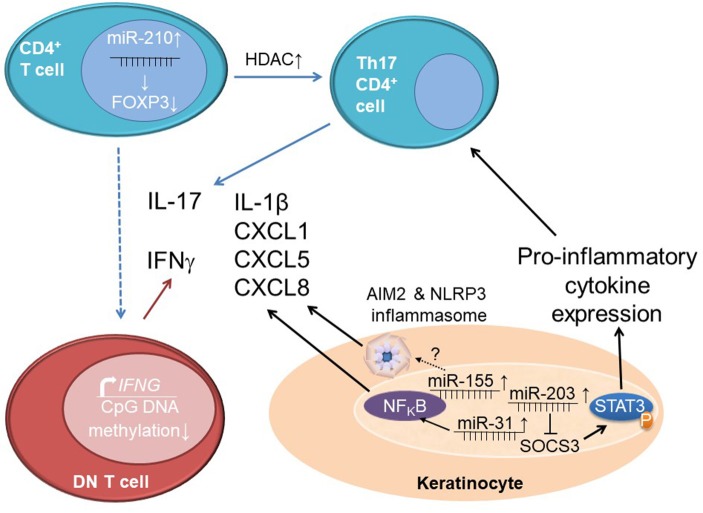
Epigenetics orchestrating interactions between immune and stroma cells in psoriasis. Increased expression of miR-210 in CD4^+^ T cells from psoriasis patients reduces the expression of the immune regulatory molecule FOXP3. Together with increased histone deacetylase (HDAC) levels that contribute to increased pro-inflammatory cytokine expression (particularly IL-17A), this leads to an imbalance between effector T cells (Th17 cell) and regulatory T cells. Effector CD3^+^CD4^−^CD8^−^ “double negative” (DN) T cells in psoriasis patients are epigenetically primed for IFN-γ expression through decreased CpG DNA methylation at a distal enhancer element of the *IFNG* gene. Keratinocytes from psoriasis patients exhibit elevated miR-203 expression which results in reduced expression of SOCS3, a negative regulator of STAT3 signaling. This contributes to increased STAT3 phosphorylation (activation) and subsequently increases pro-inflammatory cytokine expression and effector T cell differentiation. Expression of miR-31 in keratinocytes contributes to the activation of NFκB and subsequent production of IL-1β, CXCL1,-5 and-8. Elevated levels of miR-155 induce AIM2 and NLRP3 inflammasome activation through unknown mechanisms which results in enhanced IL-1β release.

Taken together, we are only beginning to understand the complex involvement of epigenetic events (see Figure 3), including DNA methylation, histone modifications and non-coding RNA expression in the mixed-pattern disease psoriasis and their involvement in the molecular pathophysiology, organ pattern, and disease progression. Even less is known about mechanisms underlying epigenetic events and which epigenetic marks are indeed disease-causing (likely T cell marks promoting effector cytokine expression) rather than effects of chronic inflammation (e.g., global DNA demethylation in skin lesions). However, current preliminary evidence is promising and warrants future studies which will deliver molecular mechanisms that may serve as biomarkers and therapeutic targets.

## Systemic Lupus Erythematosus

The systemic autoimmune/inflammatory condition SLE is frequently considered an archetypal autoimmune disease based on the presence of autoantibodies and autoreactive lymphocytes. SLE is characterized by its highly complex pathophysiology and, as a result of it, a wide range of symptoms and strong inter-individual variation, which complicates the diagnosis and treatment of this debilitating disease (4, 100, 101). However, not all patients with the diagnosis SLE (based on the clinical picture and the ACR classification criteria) indeed experience “classic” autoimmune disease. About 1–4% of all patients with SLE have monogenic conditions that are characterized by pronounced type I interferon responses, including complement deficiencies, and primary type I interferonopathies, which (at least at disease onset) much better fit the criteria of autoinflammatory diseases. While very rare, these monogenic diseases taught us much about the molecular pathophysiology of more common forms of SLE, and immune promoting, and amplifying effects of type I interferons that (other than in primary type I interferonopathies) can also be the result of tissue damage and immune complex formation. Thus, primary and secondary (as a result of tissue damage, etc.) type I IFN expression represents a strong link between innate and adaptive immune responses in the “archetypal autoimmune disease” SLE (4).

**Figure 4:**
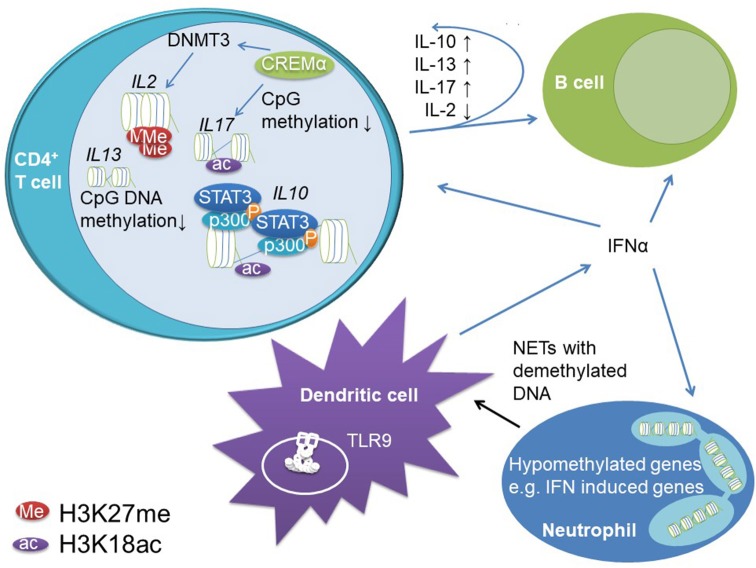
Epigenetic mechanisms contribute to dysregulation of innate and adaptive immune responses in SLE. Reduced CpG DNA methylation at *IL10* and *IL13* regulatory regions allow for increased gene expression. STAT3 recruits to *IL10* regulatory elements in the proximal promoter and an intrinsic enhancer. At these elements, STAT3 co-recruits the transcriptional co-activator p300 which has histone acetylase activity and supports chromatin decompaction through H3K18ac and increased gene expression. Increased IL-10 expression in T cells promotes B cell activity in SLE, while not affecting effector T cells (likely due to reduced IL-10 receptor expression on T cells from SLE patients). The transcription factor cAMP response element mediator (CREM)α promotes effector T cells in SLE. It induces H3K18 acetylation and CpG DNA demethylation across the *IL17* gene cluster while recruiting DNMT3 to the *IL2* locus instructing DNA methylation. Furthermore, CREMα co-recruits histone deacetylase (HDAC)1 to the *IL2* gene, which results in decreased H3K18ac and stable gene silencing. Furthermore, B and T cells are stimulated by increased type I IFN (IFN-α and –β) expression in dendritic cells and neutrophils. Neutrophils exhibit reduced CpG DNA methylation of type I IFNs and associated genes. Dendritic cells are primed for type I IFN release through stimulation of endosomal TLR9 through augmented NETosis of neutrophils. Neutrophils from SLE patients release hypomethylated DNA, which binds to TLR9 more potently when compared to methylated DNA.

The pathophysiology of SLE is incompletely understood, but strong evidence indicates the involvement of epigenetic alterations in effector lymphocyte generation, dysregulated cytokine expression and tissue damage in SLE (see Figure 4).

### Epigenetic Mechanisms Promoting Effector Lymphocytes

Epigenetic alterations have been implicated with immune cell function in SLE. First data on the involvement of epigenetic events in SLE were generated investigating DNA methylation (102). The likely strongest evidence for a causative role of DNA methylation in SLE comes from the direct comparison of PBMCs from genetically identical monozygotic twins discordant for the development of SLE, which revealed 49 regions exhibiting DNA hypomethylation in SLE patients when compared to their healthy twin (103). Explanations for decreased DNA methylation observed in immune cells from SLE patients are manifold and depend on environmental factors (UV exposure, viral infections, etc.), individual factors (genetic predisposition, age, and related enzyme activity, diet, etc.), genes investigated (hyper- vs. hypo-methylation, etc.), medication (e.g., disease-modifying antirheumatic drugs affect DNA methylation), etc. (8, 17, 104).

Effector and regulatory cytokines play a central role during immune homeostasis, lymphocyte priming, and activation. Indeed, a small study in patients with SLE refractory to standard treatment showed beneficial effects of IL-10 blocking antibodies (105). These effects have been attributed to immune activating effects of IL-10, which are involved in B cell differentiation, activation, immune globulin class switch, and the induction of immunoglobulin production (106). Indeed, IL-10 and the immune regulatory cytokine IL-13 can be measured at increased levels in the serum of SLE patients and correlate with disease activity. Increased cytokine expression was linked to DNA hypomethylation at the promoter regions of the *IL10* and *IL13* genes in CD4^+^ T cells (107). We investigated IL-10 mRNA expression in T cells from SLE patients, which was increased compared to healthy controls and correlated with disease activity. In agreement with aforementioned reports, the *IL10* promoter and in addition to this an intronic enhancer element are methylated at reduced levels in patients when compared to controls. This allows for STAT family transcription factor binding, namely STAT3 and STAT5 recruitment. In stimulated T cells from SLE patients, the activation of both elements is primarily conducted through STAT3 which replaces STAT5 at the intronic enhancer. In T cells from SLE patients STAT3 shows increased phosphorylation when compared to controls which may explain elevated recruitment to the DNA and replacement of STAT5. Furthermore, STAT proteins co-recruit the transcriptional co-activator p300 that has histone acetylase activity and confers H3K18ac at the promoter and intronic enhancer further promoting IL-10 expression (108).

Imbalanced expression of the immune regulatory cytokine IL-2 and the effector cytokine IL-17A have been centrally implicated in the pathophysiology and accrual of tissue damage in SLE. In addition to its immune promoting effects, IL-2 is required for regulatory T cell functions, and reduced IL-2 expression (as in SLE) promoted effector phenotypes (26, 109). Indeed, effector T cells are the main source of IL-17A. Altered expression of IL-2 and increased expression of IL-17A in SLE have been linked with impaired CpG DNA methylation and histone modifications in T cells from SLE patients (26, 110, 111). The transcription factor cyclic adenosine-mono-phosphate (cAMP) response element regulator (CREM)α is expressed at increased levels in T cells from patients with SLE, positively correlates with disease activity and centrally contributed to effector T cell generation and altered cytokine expression (110). CREMα co-recruits DNMT3 to the *IL2* gene resulting in CpG DNA methylation and at the same time induces DNA demethylation of *IL17A* in a yet to be determined manner. Of note, the *CREM* promoter itself is regulated by CpG DNA methylation and subject to DNA demethylation in T cells from SLE patients. Furthermore, CREMα is involved in orchestrating histone modifications in effector T cells. CD4^+^ T cells from SLE patients are characterized by increased H3K27me3 and decreased H3K18ac levels at the *IL2* promoter region as compared to healthy controls (26), which is (at least partially) mediated by the interaction between CREMα and HDAC1 at the *IL2* but not the *IL17A* promoter. Why CREMα interacts with variable epigenetic modifiers at individual promoter regions remains unknown, but the overall transcription factor environment may be involved in differential “epigenetic effects” mediated by CREMα (17). In addition to CD4^+^ effector T cells, CD4^−^CD8^−^TCR^+^CD3^+^ DN T cells are involved in the pathophysiology of SLE and characterized by high levels of IL-17A expression. DN T cells are involved in tissue damage and infiltrate the kidneys of SLE patients (112). CREMα is also centrally involved in the generation of effector DN T cells from previously CD8^+^ T cells through the down-regulation of CD8 co-receptor expression. Throughout the *CD8* cluster, CREMα co-recruits DNMT3 and histone methyltransferase G9a to regulatory regions which induces stable epigenetic silencing (77).

The most common organ complication of SLE is lupus nephritis, which can result in significant tissue damage and organ failure. The expression of miRNAs in PBMCs has been tested for its suitability as biomarker to discriminate between SLE and healthy controls, but also between active and inactive or absent lupus nephritis (113). Selected miRNAs exhibited strong differences between groups with the only limitation that subclasses of lupus nephritis could not be differentiated. The two most significantly upregulated miRNAs were miR-21 and miR-155. Of note, miR-21 was also found in psoriasis lesions (see above) and suggested to increase T cell survival in mice (93). Furthermore, miR-21 promotes activated T cell phenotypes (increased CD40L expression) and B cell differentiation through increased IL-10 expression, which also results in increased immunoglobulin production (see above). One of the targets of miR-21 is the regulatory molecule programmed cell death 4, which is encoded by *PDCD4* gene and proposed to downregulate IL-10 expression. Thus, increased miR-21 expression in SLE may resemble another contributor to previously discussed increased IL-10 levels (114). The second target identified, miR-155, can contribute to increased inflammasome activation as found in the context of psoriasis (above) through enhanced expression of NLRP3 and caspase-1 (98), thus reflecting another example for the coexistence and interconnection of autoinflammatory and autoimmune mechanisms in SLE. Furthermore, kidney biopsies allow comparison of epigenetic events in affected renal tissue and show downregulation of miR-23b and upregulation of miR-146a when compared to unaffected renal tissue from patients with kidney cancer (115). Differential miRNA expression may be the result on increased IL-17A tissue levels, since IL-17 expression mediates reduced miR-23b and increased miR-146a expression in murine fibroblasts. In HeLa cells, miR-23b inhibits IL-17A expression. Thus, miR-23b and IL-17A appear to control one another in a negative feedback loop, and reduced miR-23b levels observed in lesions may prohibit recovery. Furthermore, miR-23 suppresses NFκB activation in response to TNF-α and IL-1β through downregulation of TGF-Beta-Activated Kinase 1-Binding Protein (TAB)2, TAB3 and inhibitory κB kinase α (115). This makes miR-23 another example for closely connected innate and adaptive immune mechanisms regulated by epigenetic events in SLE.

### Epigenetic Regulation of the Innate Immune System and Type I IFN Signature

In recent years, extensive research has been undertaken focusing on type I interferons (IFN-α and IFN-β) and downstream IFN-induced gene signatures in SLE (116–118). Approximately 50% of SLE patients exhibit a type I IFN-induced gene signature in PBMCs (119), and patients with more highly elevated type I IFN-induced gene expression tend to have more severe disease manifestations, including renal, CNS and/or hematologic involvement (119). In juvenile-onset SLE patients, a pronounced type I IFN signature is present even more frequently when compared to cohorts with adult-onset disease (116). Type I IFN expression in SLE patients has been linked with the activation of plasmacytoid dendritic cells (pDCs) (120) and the presence of low-density granulocytes (LDG) (116). Since type I IFNs are potent inducers of adaptive immune responses and lymphocyte maturation and differentiation, type I IFN signatures represent a strong connection between innate and adaptive mechanisms in SLE (121).

Epigenetic events have been implicated in the increased expression of type I IFNs and associated genes. An epigenome-wide association study investigating PBMCs from SLE patients in comparison to healthy controls found 85% of differentially methylated genes to exhibit reduced CpG DNA methylation supporting findings from other studies describing low methylation levels in SLE (122). A number of genes with reduced DNA methylation were type I IFN-induced genes linking epigenetic patterns with gene expression signatures. Similar findings were reported by others, demonstrating reduced DNA methylation, and increased mRNA expression of type I IFN associated genes in B cells, T cells, monocytes and granulocytes (16). Reduced CpG DNA methylation of type I IFN associated genes is (expectedly) also present in juvenile-onset SLE (123) and contributes to increased type I IFN-induced gene expression patterns (116). Neutrophils, which exhibit even higher IFN-induced gene signatures as compared to other leukocytes (117), have 68% of their CpG sites hypomethylated and 32% hypermethylated (124). Within the hypomethylated genes, several of the most significant ones were related to type I IFN-induced genes. Interestingly, the DNA methylome of LDG was identical to the methylation pattern observed in autologous normal-density granulocytes (124), suggesting that a combination of DNA methylation and additional mechanisms may be responsible for further increased gene expression. LDGs specifically have been described to produce neutrophil extracellular traps (NETs) which lead to type I IFN release (125, 126). Since hypomethylated DNA has an increased binding capacity to TLR9 in endosomes resulting in the induction of type I interferon expression, this amplification loop may be central for maintenance of systemic inflammation in SLE (2, 127). As briefly mentioned above, several mechanisms are involved in reduced DNA methylation in immune cells from SLE patients, and include reduced activity of MAPK that contributes to reduced activity of DNMTs (128, 129). Furthermore, miRNAs have been demonstrated to negatively affect DNMTs expression in CD4^+^ T cells from SLE patients (130) linking two epigenetic events.

Upregulation of miR-126 and miR-29b in SLE patients contribute to decrease in protein levels of DNMT1 (130). miRNAs also affect type I IFN signaling in PBMCs: miR-146a is expressed at reduced levels in SLE patients which correlates with high disease activity, increased IFN scores, and proteinuria (131). Furthermore, forced expression of miR-146 reduces type I IFN induced gene expression. However, the exact underlying mechanisms and target genes remain unclear.

Taken together, the identification of a type I IFN signature in a subset of SLE patients with active disease delivered new mechanisms of amplification of inflammation. However, it currently remains unclear whether this is a primary event or rather the result of systemic inflammation and tissue damage. Indeed, it appears likely that type I IFN expression is the primary and disease-causing event only in a small subset of SLE patients with monogenic disease (namely type I interferonopathies) (4). However, it is accepted that type I interferons contribute to the maintenance and even amplify systemic inflammation and therefore may be target for therapeutic interventions. Since epigenetic events play a role in the uncontrolled expression of type I IFNs in SLE, “epigenetic interventions” may be future tools to modify disease activity and inflammation at least in a subset of patients.

## Summary and Conclusions

Epigenetic events play an impartially understood but central role in the pathophysiology of autoimmune/inflammatory conditions. The largest body of evidence exists for CpG DNA methylation, followed by histone modifications and non-coding RNAs. However, we are currently only beginning to understand that and by which mechanisms epigenetic events contribute to disease. Completely deciphering epigenetic contributors to disease is complicated by the fact that epigenetic events are highly complex and work in combination with other epigenetic marks, they are reversible and depend on multiple variables, including cell cycle, and external factors including the immunological micro-environment. While for some epigenetic modifications underlying causes and their involvement in the pathophysiology have been accepted, other modifications may be the result of ongoing inflammation and a secondary event in systemic autoimmune/inflammatory disease. Nonetheless, secondary epigenetic modifications can still alter inflammatory responses and therapeutic targeting of these may help to control inflammation and tissue damage. However, other than in cancer medicine, “epigenetic treatments” currently remain “science fiction” in the field of immunology and rheumatology, since target-directed applications are currently not available, epigenetic changes are complex, and untargeted approaches are associated with genome-wide changes that may cause significant side-effects or even worsen disease. Thus, future research is warranted to generate a more complete understanding of epigenetic contributors to inflammation and immune dysregulation, as well as their underlying molecular causes. Only a full picture of epigenetics in systemic inflammation will help us to (i) understand the exact pathophysiology of autoimmune/inflammatory conditions, (ii) deliver molecular causes for variable clinical pictures, disease severity, and outcomes in related individuals with phenotypically variable disease, and (iii) offer new targets in the search for biomarkers and individualized and target-directed treatments.

## Author Contributions

All authors listed have made a substantial, direct and intellectual contribution to the work, and approved it for publication.

### Conflict of Interest Statement

The authors declare that the research was conducted in the absence of any commercial or financial relationships that could be construed as a potential conflict of interest.
